# Low-dose hydrocortisone reduces norepinephrine duration in severe burn patients: a randomized clinical trial

**DOI:** 10.1186/s13054-015-0740-0

**Published:** 2015-01-26

**Authors:** Fabienne Venet, Jonathan Plassais, Julien Textoris, Marie-Angélique Cazalis, Alexandre Pachot, Marc Bertin-Maghit, Christophe Magnin, Thomas Rimmelé, Guillaume Monneret, Sylvie Tissot

**Affiliations:** Hospices Civils de Lyon, Cellular Immunology Laboratory, Hôpital E Herriot, Pavillon E - 5 place d’Arsonval, Lyon, Cedex 03 69437 France; Hospices Civils de Lyon, Université Claude Bernard Lyon I, Lyon EAM 4174, Lyon, 69008 France; Hospices Civils de Lyon-bioMérieux Joint Research Unit, Hôpital E Herriot, Pavillon P - 5 place d’Arsonval, Lyon, Cedex 03 69437 France; Hospices Civils de Lyon, Burn Ward, Intensive Care Unit, Hôpital E Herriot, Pavillon I - 5 place d’Arsonval, Lyon, Cedex 03 69437 France

## Abstract

**Introduction:**

The aim of this study was to assess the effect of low-dose corticosteroid therapy in reducing shock duration after severe burn.

**Methods:**

A placebo-controlled, double-blind, randomized clinical trial (RCT) was performed on two parallel groups in the burn intensive care unit (ICU). Patients were randomized to receive either low-dose corticosteroid therapy or placebo for seven days. A corticotropin test was performed at the time of randomization, before the administration of the treatment dose. Thirty-two severely burned patients with refractory shock (>0.5 μg/kg/min of norepinephrine) were prospectively included in the study.

**Results:**

We included 12 patients in the hydrocortisone-treated group and 15 patients in the placebo group in the final analysis. Among these patients, 21 were nonresponders to the corticotropin test. Median norepinephrine treatment duration (primary objective) was significantly lower in the corticosteroid-treated versus the placebo group (57 hours versus 120 hours, *P* = 0.035). The number of patients without norepinephrine 72 hours after inclusion was significantly lower in the treated group (*P* = 0.003, log-rank test analysis). The total quantities of norepinephrine administered to patients were lower in the hydrocortisone-treated versus the placebo group (1,205 μg/kg (1,079 to 2,167) versus 1,971 μg/kg (1,535 to 3,893), *P* = 0.067). There was no difference in terms of ICU or hospital length of stay, sepsis incidence, cicatrization or mortality.

**Conclusions:**

In this placebo-controlled, randomized, double-blind clinical trial, we show for the first time that the administration of low-dose hydrocortisone in burn patients with severe shock reduces vasopressor administration.

**Trial registration:**

Clinicaltrial.gov NCT00149123. Registered 6 September 2005.

**Electronic supplementary material:**

The online version of this article (doi:10.1186/s13054-015-0740-0) contains supplementary material, which is available to authorized users.

## Introduction

Severe burn injury remains a major cause of disability worldwide associated with high mortality in the case of large thermal insult (>50% total burn surface area) [1-3].

Major burn triggers a systemic inflammatory response syndrome associated with the release of circulating mediators such as histamine or cytokines. These inflammatory mediators induce similar consequences as those seen in septic shock. Indeed, after an initial hypovolemic phase, patients with extensive burn usually present a shock with increased cardiac output and reduced systemic vascular resistances [4].

In septic shock, the use of low-dose corticosteroids has been proposed as an adjunctive therapy to reduce mortality and improve shock reversal. While debate still exists regarding the impact of this treatment on septic shock-induced mortality, low-dose corticosteroids have been shown to improve systemic hemodynamics and reduce the time on vasopressor treatment in septic patients [5,6]. In addition, stress-dose hydrocortisone was suggested as a means of improving outcome in the specific subpopulation of septic patients presenting with critical-illness-related corticosteroid insufficiency [7].

Apart from some rare case reports, little is known about the influence of hydrocortisone administration in vasopressor-dependent severely burned patients with shock. Therefore, as described in septic shock, we tested the hypothesis that low-dose hydrocortisone could reduce shock duration after severe thermal injury.

Thus we designed a placebo-controlled, double-blind randomized clinical trial (RCT) to assess the effect of low-dose corticosteroid therapy in reducing shock duration after severe burn injury. In addition, a subgroup analysis was performed in patients presenting with or without relative adrenal insufficiency (RAI).

## Materials and methods

### Experimental design

**Figure 1:**
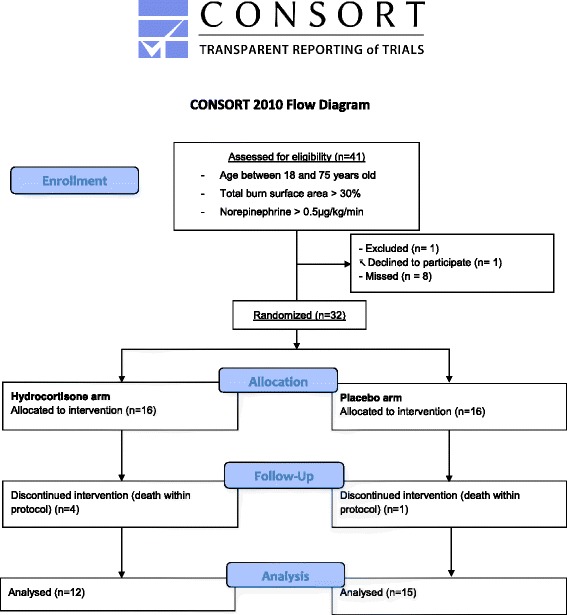
**Study flow chart.** Thirty-two patients were initially randomized in this clinical trial evaluating the effect of low-dose hydrocortisone on shock duration after severe burn injury. Inclusion and exclusion criteria are presented in the figure. At inclusion a corticotropin test was performed in every patient to identify those presenting with relative adrenal insufficiency (RAI). The follow-up duration was nine days. After this follow-up, 12 low-dose hydrocortisone-treated patients and 15 patients treated with placebo were finally included in the statistical analysis.

This placebo-controlled, randomized, double-blind study was performed on two parallel groups in the burn intensive care unit (ICU) of the university Hôpital E. Herriot, Lyon, France (Figure 1). The protocol was approved by the Comité Consultatif de Protection des Personnes dans la Recherche Biomédicale of Lyon B on 15 February 2005 and was registered at clinicaltrial.gov (NCT00149123).

### Patients

Every patient 18 years or older was prospectively enrolled in the study if meeting the following criteria: (i) age between 18 and 75 years old, (ii) total burn surface area (TBSA) over 30% of the body surface, considering only areas of second- and third-degree burns, (iii) need for norepinephrine infusion between 24 and 72 hours after the burn injury at a dose >0.5 μg/kg/min. Initial fluid resuscitation (between day (D) 0 and D2) was carried out based on the Parkland formula [8]. Initial fluid resuscitation was monitored by respiratory changes in arterial pulse pressure and by echocardiography. In cases of persistent shock after optimization of fluid resuscitation, a norepinephrine infusion was administered to maintain mean arterial pressure ≥70 mmHg.

Thirty-two severe burn patients were included between June 2005 and October 2010. Written informed consent was obtained from the patients themselves or their close relatives and a corticotropin test was performed before randomization.

Patients were not included in the case of pregnancy, other traumatic injury than burn, initial sepsis, cardiac insufficiency, contraindication or formal indication for corticosteroids or HIV-infection. Patients were excluded in the case of death during the protocol.

### Randomization

Randomization was concealed and stratified in blocks by the pharmacist. Syringes containing treatment for each patient were delivered every day to the investigator by the pharmacist following the orders of the randomization list. Every patient, medical and nursing staffs remained blinded throughout the study period.

### Treatments

The treated group received hydrocortisone intravenously as follows: a priming dose of 50 mg of hydrocortisone (Upjohn, Serb Laboratoire, Paris, France) followed by a continuous infusion of 200 mg/day hydrocortisone dissolved in physiological saline for five days, 100 mg the sixth day and 50 mg the seventh day. Placebo (NaCl 0.9% solution) infusions were indiscernible from active treatments. Treatment duration was seven days. Every patient was under mechanical ventilation. Sedation was administered according to a nurse-driven algorithm to achieve a Ramsay scale score of between 3 and 4 [9].

### Data collection at inclusion

#### Clinical evaluation

The following data were recorded: (i) demographic characteristics including age, gender, usual weight and weight at inclusion, (ii) severity of illness assessed by TBSA (according to the Lund and Browder tables), Baux and Abbreviated Burn Severity Index (ABSI) scores, inhalation injury, fraction of inspired oxygen (FiO2) and positive end-expiratory pressure (PEEP) at inclusion (iii) interventions including number of skin grafts, etomidate administration prior inclusion, norepinephrine dosage and number of blood transfusions.

#### Laboratory variables

Hematological and chemistry data and blood gas analysis were determined before inclusion. A corticotropin test was performed using a 250 μg intravenous bolus of tetracosactin (Synacthen™, Ciba-Geigy, Rueil-Malmaison, France) [10]. Blood samples were taken immediately before the test and 60 minutes afterward and plasma were stored at -80°C until assayed. Cortisol was measured blindly and serially before the final statistical analyses. RAI was defined by a baseline cortisol of less than 10 μg/dl or a delta cortisol of less than 9 μg/dl [11].

### Follow-up

The follow-up duration of hemodynamic instability was nine days after inclusion in the protocol (that is until two days after the end of treatment). Quantity and duration of norepinephrine treatment and development of septic shock were monitored during the follow-up. The occurrence of death and secondary infections during 28 days after protocol inclusion was recorded.

### End points

The primary end point was duration of shock from randomization to the end of the follow-up. This was evaluated by the norepinephrine treatment duration (in hours) after inclusion in the protocol. Secondary end points were the proportion of patients without norepinephrine at 72 hours after inclusion, total amount of norepinephrine, ICU and hospital length of stay, sepsis incidence, number of skin grafts, and mortality at day 28.

### Sample size and statistical analysis

Seventeen patients per group was the calculated sample size needed, in a two-sided *t* test performed with a 0.05 type 1 error and a power of 0.80, to detect a difference of 48 hours between the two groups for patients alive after seven days of treatment [12]. However, due to the extended duration of patient recruitment (five years for 32 patients), this study was prematurely ended by the promoter (Hospices Civils de Lyon).

For continuous variables, medians [interquartile ranges Q1 to Q3] are reported. For categorical variables, the number of patients in each category and the corresponding percentages are given. Pretreatment clinical and biological characteristics were compared between groups using Mann-Whitney tests (continuous variable) or Fisher’s exact tests when appropriate (categorical variables).

Regarding primary outcome analysis, the differences in norepinephrine duration and dosage during the complete follow-up were compared using Mann-Whitney tests. A log-rank test analysis was performed to compare the probability of norepinephrine duration in treated and non-treated patients during the first 72 hours after inclusion in the protocol.

Early deaths were excluded from the analysis because their inclusion would have overestimated the difference in vasopressor treatment duration between the groups. In addition, statistical analyses that may consider early deaths could not be performed because of the low number of patients included in the RCT.

*Post hoc* power analysis were performed with *pwd* R package (effect size = 1.07). The statistical analyses were performed using R (version 3.0.0 (2013-04-03)) and *P* values were considered significant when lower than 0.05.

## Results

### Study description

From June 2005 to October 2010, 32 severe burn patients that developed shock (norepinephrine >0.5 μg/kg/min) were included in the study (Figure 1). Inclusion and exclusion criteria are reported. During the clinical follow-up, four patients in the hydrocortisone-treated group died and one patient died in the placebo group. Therefore, 12 patients in the hydrocortisone-treated group and 15 patients in the placebo group were included in the final analysis (Figure 1). Among these patients, 21 were nonresponders to corticotropin test; that is presented with RAI (placebo, n = 12; corticosteroids, n = 9).

### Characteristics of burn patients at inclusion

Individual clinical data for the 27 patients are presented in Table S1 in Additional file 1. Grouped clinical characteristics are reported on Table 1. At baseline, the two groups were overall balanced with respect to general characteristics and severity of illness. TBSA ranged from 30% and 95% and the median value was 62%. ABSI ranged from 8 to 14 (median = 11) and Baux index from 81 to 146 (median = 108). There were no differences between the two groups concerning these burn severity indexes, and the number of RAI. Etomidate injection prior to inclusion in the protocol was significantly more frequent in the placebo group (80%) compared with the corticosteroid-treated group (27%, *P* = 0.015). Inhalation injuries were more frequent in the hydrocortisone-treated group (58%) as compared with placebo group (20%, *P* = 0.057).

**Table 1:** **Demographic and clinical characteristics of 27 burn patients**

	**Corticosteroids (n = 12)**	**Placebo (n = 15)**	**Total (n = 27)**	***P*** **value**
**Demographic characteristics**				
Age (years)	48 [46-50]	48 [36-58]	48 [39-54]	0.90
Gender (males)	10 (83%)	9 (60%)	19 (70%)	0.24
Weight (usual), kg	73 [69-83]	80 [68-93]	76 [68-85]	0.57
Weight at inclusion, kg	92 [80-96]	100 [81-112]	94 [80-103]	0.22
**Severity**				
Total burn surface area (%)	66 [50-85]	62 [44-76]	62 [47-81]	0.31
Baux score	115 [101-128]	108 [100-113]	108 [100-123]	0.35
ABSI score	12 [11-13]	11 [10-12]	11 [10-12]	0.19
**Clinical characteristics prior inclusion**				
Delay burn - inclusion (h)	60 [54-65]	45 [41-58]	54 [43-62]	**0.03**
Inhalation injury	7 (58%)	3 (20%)	10 (37%)	**0.06**
Etomidate administration	3 (27%)	12 (80%)	15 (58%)	**0.02**
Norepinephrine (μg/kg/min)	0.57 [0.51-0.62]	0.60 [0.51-0.92]	0.60 [0.50-0.70]	0.45
Blood transfusions	1 (8%)	2 (13%)	3 (11%)	1.00
FiO2 (%)	40 [30-45]	40 [40-50]	40 [35-50]	0.13
PEEP (cmH_2_O)	4 [4,5]	6 [5-7]	5 [4-6]	0.17
Diuresis (mL/24 h)	3,925 [3,175-4,400]	3,500 [3,050-4,250]	3,600 [3,150-4,400]	0.59
**Biology**				
Relative adrenal insufficiency	9 (75%)	12 (80%)	21 (78%)	1.00
Basal cortisol (μg/dL)	15 [9-18]	11 [8-16]	14 [8-17]	0.39
Delta cortisol after-before corticotropin test (μg/dL)	12 [7-17]	9 [5-13]	10 [6-14]	0.32
Plasma creatinine (μmol/L)	78 [75-91]	92 [72-103]	80 [73-103]	0.68
Plasma protein (g/L)	43 [38-46]	41 [38-45]	42 [38-45]	0.71
Plasma albumin (g/L)	29 [27-31]	29 [28-31]	29 [27-31]	0.86
Hemoglobin (g/L)	111 [104-120]	113 [90-132]	113 [100-125]	0.98
WBCC (10^9^/L)	4 [3-7]	7 [4-11]	6 [3-10]	0.13
Lymphocyte count (10^9^/L)	0.85 [0.61-1.25]	1.15 [0.92-1.43]	1 [0.72-1.37]	0.15
**RCT end points**				
Norepinephrine duration (h)	57 [38-113]	120 [84-141]	96 [57-129]	**0.03**
Norepinephrine quantity (μg/kg)	1,205 [1,079-2,167]	1,971 [1,535-3,893]	1,755 [1,174-3,188]	0.07
Septic shock	4 (33%)	7 (47%)	11 (41%)	0.70
Infections per patient	2 [1-3]	2 [2,3]	2 [2-3]	0.43
Skin grafting per patient	6 [4-7]	5 [4-10]	6 [4-9]	0.45
Stay in ICU (days)	63 [27-77]	62 [41-81]	62 [39-78]	0.69
Stay in hospital (days)	73 [54-85]	84 [58-104]	75 [58-97]	0.45
Death before D28	6 (38%)	2 (13%)	8 (25%)	0.57

Demographic and clinical characteristics of 27 severe burn patients (12 patients with low-dose of hydrocortisone (corticosteroids) and 15 patients with placebo (placebo)). Data reported in the second and third column are the medians and the interquartile ranges between brackets or percentages between parentheses. Patient’s characteristics were compared between groups using Mann-Whitney tests (continuous variable) or Fisher’s exact tests when appropriate (categorical variables). ABSI, Abbreviated Burn Severity Index; h, hours; FiO2, fraction of inspired oxygen; PEEP, positive end-expiratory pressure; WBCC, white blood cell count; RCT, randomized clinical trial; ICU, intensive care unit.

### Norepinephrine dosage and duration

**Figure 2:**
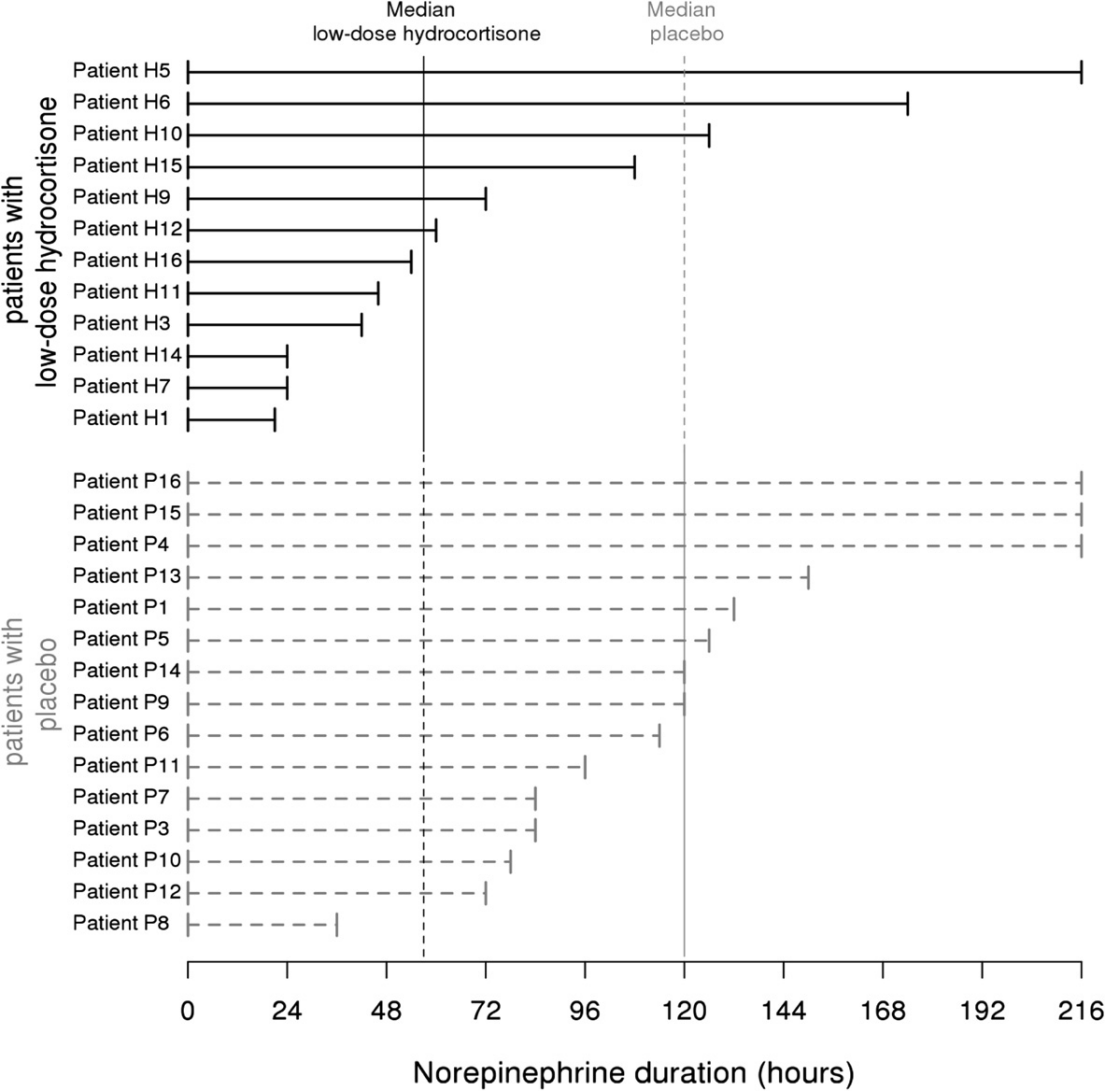
**Individual longitudinal follow-up plots of vasopressor therapy.** Vasopressor therapy duration is presented in this figure for the analyzed patients (n = 27). The dashed lines correspond to norepinephrine treatment duration for patients with placebo and the solid lines correspond to norepinephrine treatment duration for patients with corticosteroids. Medians of norepinephrine duration are reported (57 hours in the hydrocortisone-treated group versus 120 hours in the placebo-treated group).

We then analyzed norepinephrine treatment in placebo or low-dose corticosteroid-treated patients (Figure 2). Prior inclusion in the protocol, the quantities of injected norepinephrine between the treated and non-treated groups were identical (median value: 0.60 μg/kg/min per patients, *P* = 0.445, Table 1). However, total quantities of norepinephrine administered to patients during the protocol were lower in the hydrocortisone-treated vs the placebo group (1,205 μg/kg [1,079 to 2,167] vs 1,971 μg/kg [1,535 to 3,893], *P* = 0.067, Table 1).

**Figure 3:**
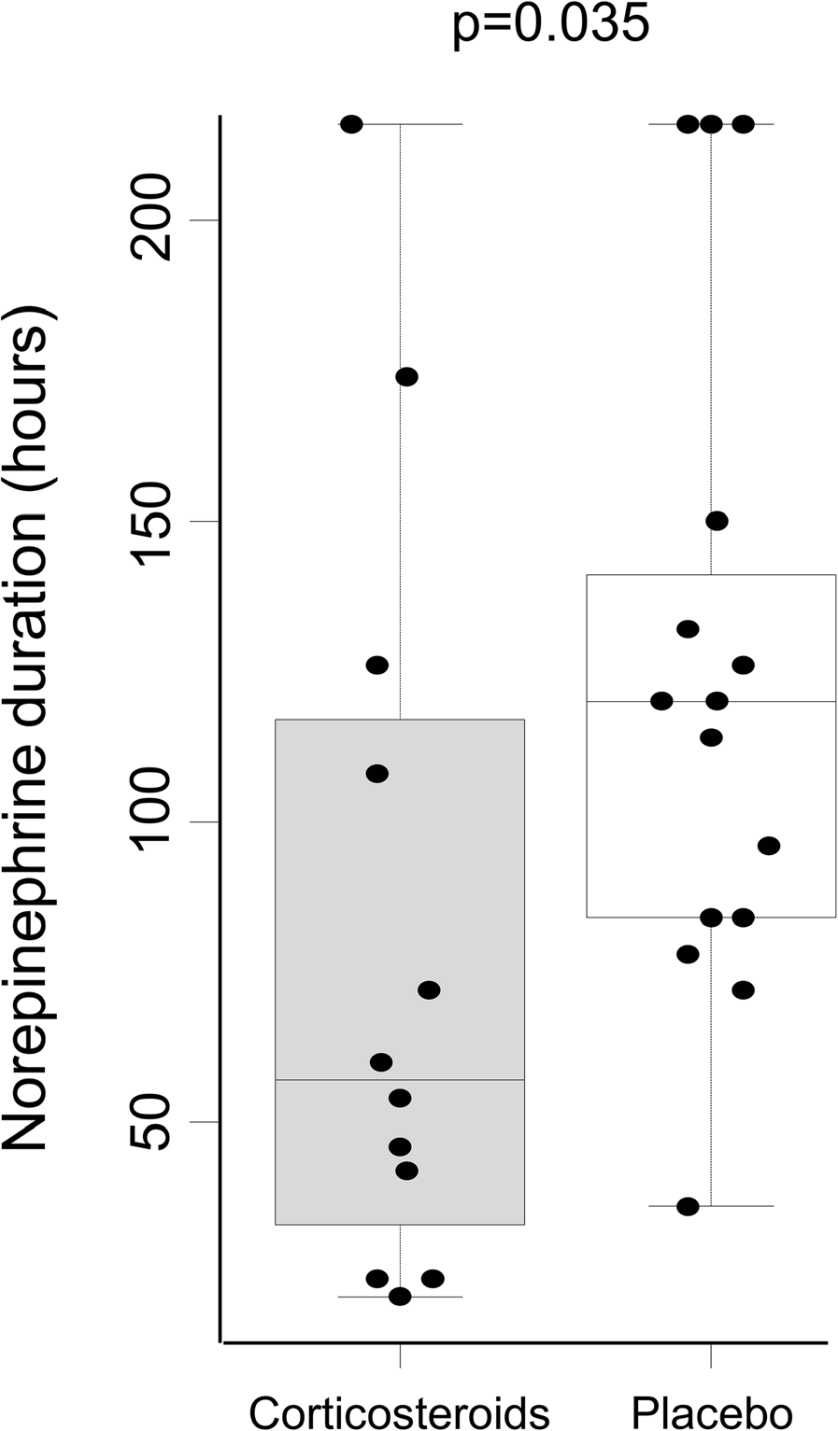
**Norepinephrine duration for patients treated with low-dose hydrocortisone and placebo.** Thirty-two severely burned patients were initially randomized in this clinical trial evaluating the effect of low-dose hydrocortisone on shock duration after burn. Twelve low-dose hydrocortisone-treated patients (corticosteroids, grey boxes) and 15 patients treated with placebo (placebo, open boxes) were finally included in the statistical analysis. Box plots and individual values of norepinephrine duration (in hours) in low-dose hydrocortisone and placebo-treated groups are presented. Differences between groups were evaluated by using the Mann-Whitney test.

**Figure 4:**
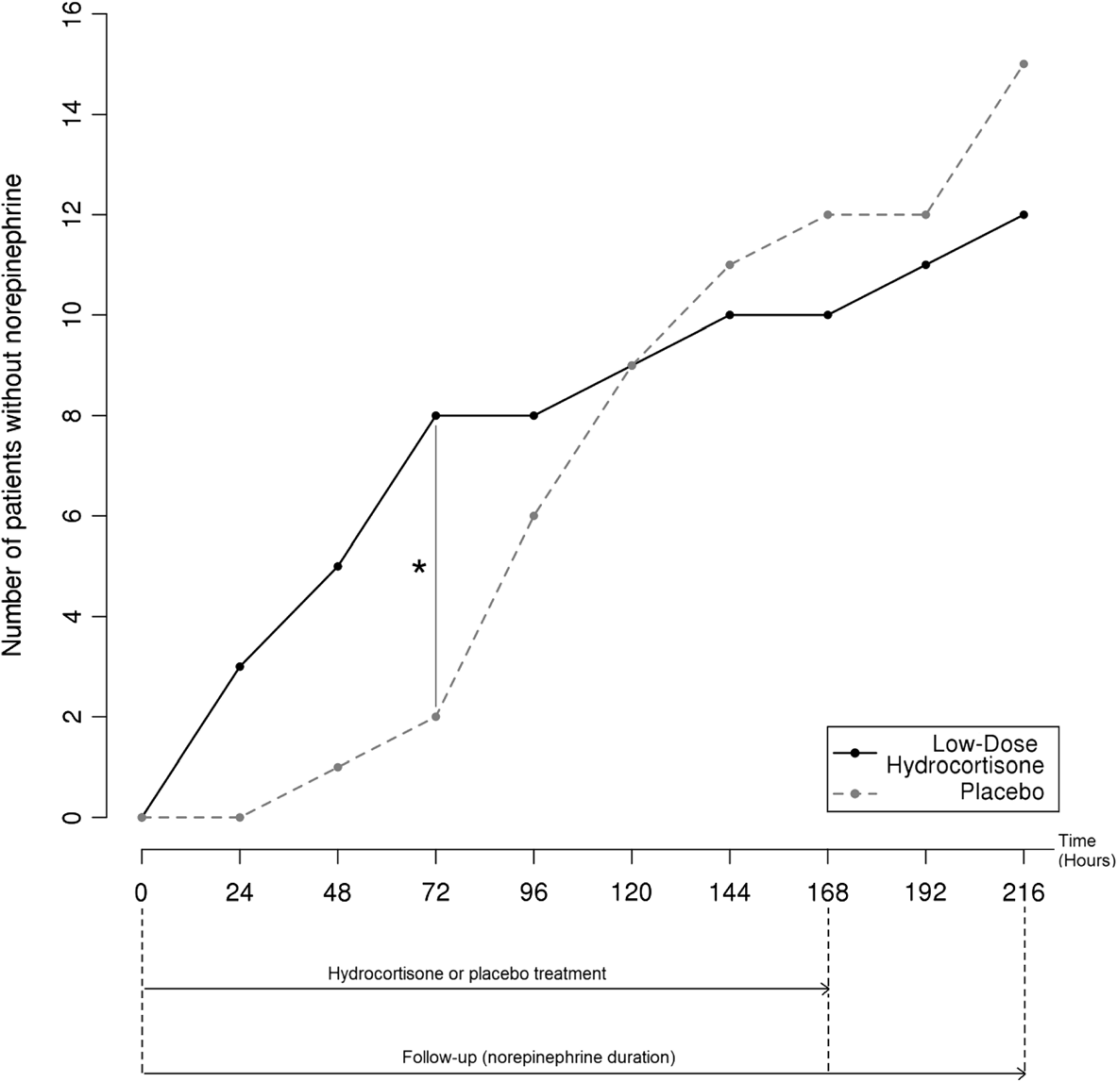
**Number of patients without norepinephrine at nine time points during the follow-up.** Thirty-two severely burned patients were initially randomized in this clinical trial evaluating the effect of low-dose hydrocortisone on shock duration after burn. Twelve low-dose hydrocortisone-treated patients and 15 patients treated with placebo were finally included in the statistical analysis. The numbers of patients without norepinephrine at nine time points after protocol inclusion are shown. The solid black line corresponds to patients treated with low-dose hydrocortisone. The dashed grey line corresponds to patients treated with placebo. Low-dose hydrocortisone or placebo treatments and follow-up durations are shown. Seventy-two hours after inclusion, the difference of weaned patients between the two groups was evaluated with a Fisher’s exact test.

Interestingly, median norepinephrine treatment duration was significantly lower in the corticosteroid-treated vs the placebo group (57 hours vs 120 hours, *P* = 0.035, Figures 2 and 3). Although the number of samples according to the sample size calculation was not reached, the power estimated for this test between the two groups is 0.76. The number of patients without norepinephrine 72 hours after inclusion was significantly lower in the treated group (*P* = 0.003, log-rank test analysis). Indeed, only two placebo patients (13%) were weaned of norepinephrine in the placebo group against eight patients in the low-dose hydrocortisone treated group (67%, *P* = 0.007, Fisher’s exact test, Figure 4). Most patients were nonresponders to the corticotropin test (n = 21, 78%). A descriptive analysis of the treatment effect was performed in each subgroup (Table S2 in Additional file 1: supplemental digital content 2). Due to the very low number of patients in the responder group, we cannot conclude about norepinephrine dosage and duration between these two groups of patients. However, a similar trend toward an earlier weaning of vasopressors was observed in both groups.

## Discussion

Burn injury triggers a systemic inflammatory response syndrome that participates in a severe cardiovascular dysfunction called burn shock. Indeed, widespread skin destruction induces a large necrotic mass that leads to an intense inflammatory reaction. This activates keratinocytes, endothelial cells and recruits neutrophils. Certain mediators (endothelin, histamine, bradykinins, serotonin and so on) are released in large quantities and act both at the site of burn and at distance. This results in hypovolemia associated with hemoconcentration, hyponatremia, hypoalbuminemia and myocardial malfunction. This hypovolemia can rapidly become irreversible if fluids are not administered [4]. Importantly, it has been shown that shock severity and duration are important surrogate markers of mortality after severe burn injury.

Current treatment of burn shock comprises both vasopressor agents and volume therapy [4]. However, any intervention strategy that is capable of reducing dose rates of vasoconstrictors as well as reducing volume substitution will likely improve prognosis and outcome of severe burn patients [13]. However, the need for vasopressor support prevents the transfer out of the critical care setting. This has implications both for the patients (reduced risk of iatrogenic adverse events) and for the health-care system (decreased costs and better allocation of scarce resources). Thus earlier reversal of shock is a clinically relevant end point to consider and may be a worthwhile goal after severe burn injury.

Low-dose hydrocortisone therapy may represent one such treatment. Indeed, this treatment has been shown to improve rapidly (1 hour after treatment administration) vascular responsiveness to catecholamines in septic shock patients and healthy volunteers [14,15]. In septic shock patients, seminal studies by Annane *et al*. [7] showed that low-dose hydrocortisone therapy reduced mortality and duration of vasopressor administration in these patients. Although the effect of low-dose corticosteroid therapy on septic shock mortality has been challenged within recent years, the effect of this treatment on shock reversal (duration of vasopressor treatment or cumulative dosages) has been confirmed by several meta-analyses. Indeed, in a meta-analysis published in 2012 for the American Academy of Emergency Medicine, Sherwin *et al*. [16] reported that, of the seven clinical trials they identified testing low-dose corticosteroids in septic shock patients, all seven trials reported shock reversal or the withdrawal of vasopressors. These seven trials included 1,005 patients with 505 and 500 patients in the intervention and control arms respectively. Pooled results revealed that the relative risk of shock reversal was 1.17 (95% confidence interval 1.07 to 1.28). Similarly, in a meta-analysis of RCTs from 1997 to 2008, Minneci *et al.* [6] reported that low-dose steroids demonstrated a consistent improvement in shock reversal across the trials reporting this outcome. Finally, Sligl *et al.* [5] identified six studies from 1998 to 2008 reporting appropriate data on this aspect. They observed a statistically significant difference in the incidence of shock reversal at seven days between the group that received corticosteroids and the control group. Subgroup analysis of four studies examining shock reversal in regard to adrenal responsiveness showed statistical significant effects in both responders and nonresponders. This demonstrates that, after septic shock, low-dose corticosteroids do reverse shock faster, therefore freeing valuable resources in the ICU.

Therefore, we designed this placebo-controlled, randomized, double-blind clinical trial to test the hypothesis that low-dose corticosteroid therapy may reduce shock duration after severe thermal injury. Only few studies and case reports investigated the influence of low-dose hydrocortisone administration in vasopressor-dependent burn patients. In a case report published in 2002, Nácul *et al.* [17] reported that a three-day steroid treatment in a 39-year-old severely burned patient (80% TBSA) with hemodynamic instability and low response to intravenous fluid or vasopressors for 20 days in the ICU, led to blood pressure normalization without the administration of any vasopressor. James *et al*. [18] demonstrated the beneficial role of hydrocortisone in a 75% burned patient with long-standing Addison disease. Similarly, in a prospective study including 14 consecutive severely burned patients with septic shock, Winter *et al.* [19] observed that low-dose hydrocortisone therapy was associated with a significant reduction in norepinephrine dosages in surviving patients in association with a significant reduction in median fluid requirement. Finally, in a retrospective study including 19 burn patients with septic shock, Fuchs *et al.* [20] showed a beneficial effect on both morbidity and mortality of low-dose hydrocortisone therapy compared with control patients. In this study, the median total time application of vasopressors was significantly reduced in the treated group of patients. Therefore, these preliminary data showed overall a beneficial effect of low-dose corticosteroid therapy on burn shock.

In line, our study is the first prospective randomized placebo-controlled clinical trial to show that low-dose hydrocortisone significantly reduces vasopressor administration at 72 hours in severely burned patients. Indeed, we observed that median time to vasopressor therapy withdrawal was shorter in the hydrocortisone-treated group (57 hours) versus the placebo-treated group (120 hours) and that the norepinephrine treatment duration was significantly lower in the corticosteroid-treated groups than the placebo group (Mann-Whitney test: *P* = 0.036). These results are in accordance with previous studies in septic shock patients. For example, a single intravenous administration of 50 mg of hydrocortisone was shown to strongly improve norepinephrine and phenylepinephrine mean arterial pressure dose-response relationships in patients with septic shock, especially in those with RAI [14].

The mechanisms underlying this effect are unclear but an improved vasopressor responsiveness of peripheral vessels may play an important role. Indeed, hydrocortisone raises blood volume, increases vascular tone and enhances endothelial reactivity to vasopressors [21]. Similarly, cortisol plays an important role in maintaining of vascular tone and myocardial contractility and has an important permissive effect on the action of catecholamine on vascular smooth muscle [22]. In addition, at stress doses, corticosteroids have been shown to increase neutrophil activity, increase the homing of dendritic cells with preservation of monocyte and interleukin 12 (IL-12) functions, and attenuate the overwhelming inflammatory response that may lead to shock after severe burn injury.

An additional interesting result of our RCT is to show that low-dose corticosteroids may have such an effect in both responders and nonresponders to the corticotropin test. Indeed, we observed that norepinephrine was withdrawn earlier in the hydrocortisone-treated group (median norepinephrine duration = 60 hours) than in the placebo group (117 hours) of patients with relative corticosteroid insufficiency (Figure S1 in Additional file 1), but it was also the case in responder patients (median norepinephrine duration = 42 hours in the treated patients, vs 132 hours in the placebo group, Table S2 in Additional file 1). Although the low number of patients implies this result to be confirmed, our data overall suggest that the corticotropin test may not be necessary to guide low-dose hydrocortisone replacement therapy in burn patients with severe shock.

This study has some limitations. First, the 0.5 μg/kg/min vasopressor inclusion criteria led to a slow accrual in the study. Therefore, we did not reach the sample size calculated before the start of the clinical trial. This consequently decreased the statistical power of our study and we were unable to reach statistical significance in some of our analyses (RAI subgroup analysis). Second, we did not perform an intention-to-treat analysis because the inclusion of early deaths would have overestimated the difference in vasopressor treatment duration between the groups. In addition statistical analyses that may consider early deaths could not be performed because of the low number of patients included in the RCT. Third, the number of deaths is higher in the hydrocortisone group as compared with placebo-treated patients. This has to be put into perspective with studies in septic shock that showed an increased mortality in low-dose hydrocortisone-treated patients [16]. In addition, it is to note that inhalation injuries were more frequent in the treated versus the placebo group and that this may have participated in this higher mortality. Fourth, etomidate treatment was significantly different between groups (*P* = 0.02, Fisher’s exact test, Table 1). However, as etomidate injections have been shown to block cortisol synthesis and as this treatment was more frequent in the placebo group than in the hydrocortisone-treated group, this bias has to be taken into account in the interpretation of the report. In addition, this potential bias is supported by the observation of Vinclair *et al*. that one single etomidate injection upon initial intubation had a negative effect on cortisol synthesis for 48 hours [23]. However, whether this effect may occur in the particular context of burn patients remains to be investigated. Therefore, considering these limitations, we want to emphasize that results presented in this study are only preliminary and need to be confirmed through an intention-to-treat, multicentric RCT including a larger cohort of patients.

## Conclusions

In this placebo-controlled, double-blind RCT we show for the first time that the administration of low-dose hydrocortisone in burn patients with severe shock reduces vasopressor administration. Indeed, in the treated group of patients, we observed a significant withdrawal of vasopressor therapy within the first 72 hours after inclusion. This effect was present in burn patients with or without RAI. Our positive results support a larger clinical trial in order to confirm the positive effect of low-dose hydrocortisone on reducing vasopressor administration after severe burn and to assess the potential beneficial effects of weaning vasopressors earlier in these patients.

## Key messages

- This study is the first placebo-controlled, double-blind, randomized clinical trial testing the effect of low-dose corticosteroid therapy in reducing shock duration after severe burn injury.
- Median norepinephrine treatment duration (primary objective) was significantly lower in the corticosteroid-treated vs placebo group (57 hours vs 120 hours, *P* = 0.035).
- The number of patients without norepinephrine 72 hours after inclusion was significantly lower in the treated group (*P* = 0.003, log-rank test analysis).
- The total quantities of norepinephrine administered to patients were lower in the hydrocortisone-treated vs placebo group (1,205 μg/kg [1,079 to 2,167] vs 1,971 μg/kg [1,535 to 3,893], *P* = 0.067).
- In this placebo-controlled, randomized, double-blind clinical trial, we show for the first time that the administration of low-dose hydrocortisone in burn patients with severe shock reduces vasopressor administration.

## Additional file

Additional file 1: Table S1. Individual demographic and clinical characteristics of the 32 burn patients. **Table S2.** Demographic and clinical characteristics of the burn patients with or without RAI. **Figure S1.** Norepinephrine duration for nonresponder patients treated with low-dose hydrocortisone and placebo.

## Abbreviations

ABSI: Abbreviated Burn Severity Index
D: day
FiO2: fraction of inspired oxygen
ICU: intensive care unit
IL-12: interleukin 12
PEEP: positive end-expiratory pressure
RAI: relative adrenal insufficiency
RCT: randomized clinical trial
TBSA: total burn surface area
