# Supplementary material for: Low-dose hydrocortisone reduces norepinephrine duration in severe burn patients: a randomized clinical trial
Source: Crit Care. 2015 Jan 26;19(1):21. doi: 10.1186/s13054-015-0740-0 (PMC4347659; doi:10.1186/s13054-015-0740-0)

**Additional file 1**

**Table S1 Individual demographic and clinical characteristics of the 32 burn patients.**

Treatment group 1= Low-dose hydrocortisone, Treatment group 2= placebo. RAI= Relative adrenal insufficiency. Norep= Norepinephrine. Eto= Etomidate treatment. ABSI= Abbreviated Burn Severity Index. TBSA= Total Burn Surface Area

| **Patient** | **Treatment**  **Group** | **RAI**  **(Y/N)** | **Sex (M/F)** | **Age** | **TBSA (%)** | **Baux Index** | **ABSI** | **Inhalation injury (Y/N)** | **Eto** | **Death during shock phase** | **Norep duration (hours)** |
| --- | --- | --- | --- | --- | --- | --- | --- | --- | --- | --- | --- |
| H1 | 1 | No | M | 47 | 50 | 97 | 9 | Yes | No | No | 21 |
| H2 | 1 | No | M | 41 | 46 | 87 | 10 | Yes | No | Yes | 174 |
| H3 | 1 | No | M | 36 | 95 | 131 | 13 | No | No | No | 42 |
| H4 | 1 | No | F | 48 | 64 | 112 | 13 | Yes | No | Yes | 150 |
| H5 | 1 | No | M | 46 | 57 | 103 | 11 | Yes | No | No | 216 |
| H6 | 1 | Yes | F | 49 | 35 | 84 | 9 | Yes | No | No | 174 |
| H7 | 1 | Yes | M | 50 | 57 | 107 | 11 | Yes | - | No | 24 |
| H8 | 1 | Yes | M | 19 | 98 | 117 | 12 | No | Yes | Yes | 108 |
| H9 | 1 | Yes | M | 50 | 49 | 99 | 9 | No | No | No | 72 |
| H10 | 1 | Yes | M | 46 | 81 | 127 | 13 | No | Yes | No | 126 |
| H11 | 1 | Yes | M | 52 | 85 | 137 | 14 | Yes | Yes | No | 46 |
| H12 | 1 | Yes | M | 48 | 75 | 123 | 12 | No | No | No | 60 |
| H13 | 1 | Yes | M | 42 | 88 | 130 | 14 | Yes | Yes | Yes | 42 |
| H14 | 1 | Yes | F | 64 | 38 | 102 | 11 | Yes | Yes | No | 24 |
| H15 | 1 | Yes | M | 49 | 90 | 139 | 14 | Yes | No | No | 108 |
| H16 | 1 | Yes | M | 40 | 85 | 125 | 12 | No | No | No | 54 |
| P1 | 2 | No | M | 34 | 47 | 81 | 9 | Yes | Yes | No | 132 |
| P2 | 2 | No | F | 58 | 70 | 128 | 12 | No | Yes | Yes | 192 |
| P3 | 2 | No | F | 56 | 30 | 86 | 8 | No | No | No | 84 |
| P4 | 2 | No | M | 56 | 90 | 146 | 13 | No | Yes | No | 216 |
| P5 | 2 | Yes | M | 38 | 70 | 108 | 10 | No | Yes | No | 126 |
| P6 | 2 | Yes | M | 66 | 35 | 101 | 9 | No | No | No | 114 |
| P7 | 2 | Yes | M | 35 | 88 | 123 | 12 | No | Yes | No | 84 |
| P8 | 2 | Yes | F | 75 | 40 | 115 | 10 | No | Yes | No | 36 |
| P9 | 2 | Yes | M | 25 | 78 | 103 | 11 | No | Yes | No | 120 |
| P10 | 2 | Yes | M | 36 | 73 | 109 | 12 | Yes | Yes | No | 78 |
| P11 | 2 | Yes | F | 59 | 38 | 97 | 9 | No | Yes | No | 96 |
| P12 | 2 | Yes | F | 32 | 70 | 102 | 11 | No | Yes | No | 72 |
| P13 | 2 | Yes | M | 48 | 62 | 110 | 11 | Yes | Yes | No | 150 |
| P14 | 2 | Yes | F | 63 | 47 | 110 | 11 | No | Yes | No | 120 |
| P15 | 2 | Yes | M | 43 | 80 | 123 | 12 | No | No | No | 216 |
| P16 | 2 | Yes | F | 51 | 47 | 98 | 11 | No | Yes | No | 216 |

**Table S2 Demographic and clinical characteristics of the burn patients with or without RAI.**

Demographic and clinical characteristics of 27 severe burn patients, 21 of which were non-responders to corticotropin test (9 patients with low-dose of hydrocortisone and 12 patients with placebo) and 6 of which were responders (3 patients with low-dose of hydrocortisone and 3 patients with placebo). Data reported are the medians and the interquartile ranges between brackets or percentages between parentheses. ICU= Intensive Care Unit, RCT= Randomized Clinical Trial, RAI= Relative Adrenal Insufficiency, PEEP= Positive end-expiratory pressure, h = hours, nbr = number, WBCC = white blood cell count.

|  | **Non-responders to corticotropin test** | | | **Responders to corticotropin test** | | |
| --- | --- | --- | --- | --- | --- | --- |
|  | **Corticosteroids**  **(n=9)** | **Placebo**  **(n=12)** | **Total (n=21)** | **Corticosteroids**  **(n=3)** | **Placebo**  **(n=3)** | **Total (n=6)** |
| **Demographic characteristics** | | | | | | |
| Age (years) | 49 [48-50] | 46 [36-60] | 49 [40-52] | 46 [41-47] | 56 [45-56] | 47 [39-56] |
| Gender (males) | 7 (78%) | 7 (58%) | 14 (68%) | 3 (100%) | 2 (66.67%) | 5 (83%) |
| Weight (usual), kg | 70 [65-83] | 80 [69-89] | 80 [66-85] | 73 [73-75] | 80 [68-95] | 75 [72-79] |
| Weight at inclusion, kg | 90 [75-95] | 97 [82-105] | 93 [78-102] | 95 [88-96] | 115 [96-123] | 96 [85-111] |
| **Severity** | | | | | | |
| Total burn surface area (%) | 75 [49-85] | 66 [45-74] | 70 [47-80] | 57 [54-76] | 47 [39-69] | 54 [48-82] |
| Baux score | 123 [102-127] | 109 [102-111] | 109 [102-123] | 103 [100-117] | 86 [84-116] | 100 [89-124] |
| ABSI score | 12 [11-13] | 11 [10-11] | 11 [10-12] | 11 [10-12] | 9 [9-11] | 10 [9-13] |
| **Clinical Characteristics prior inclusion** | | | | | | |
| Delay burn- inclusion, h | 62 [57-64] | 43 [40-48] | 51 [41-58] | 54 [52-63] | 61 [61-66] | 61 [56-69] |
| Inhalation injury (%) | 5 (56%) | 2 (17%) | 7 (34%) | 2 (67%) | 1 (34%) | 3 (50%) |
| Etomidate administration (%) | 3 (38%) | 10 (83%) | 13 (65%) | 0 (0%) | 2 (67%) | 2 (33%) |
| Norepinephrine, µg/kg/min | 0.59 [0.51-0.61] | 0.60 [0.52-0.86] | 0.60 [0.51-0.65] | 0.55 [0.52-0.74] | 0.70 [0.60-0.87] | 0.62 [0.51-0.86] |
| Blood transfusions | 1 (11%) | 1 (8%) | 2 (10%) | 0 (0%) | 1 (33%) | 1 (17%) |
| FiO2, % | 40 [30-40] | 40 [39-50] | 40 [35-45] | 50 [40-60] | 60 [55-60] | 55 [50-60] |
| PEEP, cmH2O | 4 [4-5] | 5 [5-6] | 5 [4-6] | 3 [3-6] | 8 [8-9] | 8 [4-8] |
| Diuresis, ml/24h | 4300 [3600-4400] | 3358 [2825-4050] | 3600 [3100-4400] | 3200 [1810-3450] | 4000 [3675-4500] | 3525 [3238-3925] |
| **Biology** |  |  |  |  |  |  |
| Basal cortisol (μg/dl) | 10 [8-15] | 9 [8-14] | 10 [8-15] | 24 [20-27] | 20 [17-25] | 22 [16-28] |
| Delta cortisol after-before synacten test (μg/dl) | 9 [6-13] | 7 [5-11] | 7 [5-13] | 28 [23-30] | 13 [12-14] | 16 [13-26] |
| Plasma creatinine (µmol/L) | 78 [70-78] | 90 [74-101] | 78 [70-93] | 125 [103-154] | 99 [75-134] | 112 [85-157] |
| Plasma protein (g/L) | 42 [38-44] | 42 [38-44] | 42 [38-44] | 44 [40-50] | 40 [40-47] | 42 [40-51] |
| Plasma albumin (g/L) | 29 [27-31] | 30 [28-31] | 29 [28-31] | 29 [28-36] | 27 [25-32] | 28 [26-35] |
| Hemoglobin (g/L) | 109 [105-119] | 112 [89-130] | 111 [99-126] | 115 [109-120] | 113 [101-135] | 114 [105-122] |
| WBCC (10^9^/L) | 4 [3-8] | 8 [5-11] | 7 [3-10] | 5 [3-6] | 6 [4-10] | 6 [2-6] |
| Lymphocyte count (10^9^/L) | 0.80 [0.61-1.40] | 1.30 [1.00-1.69] | 1.06 [0.80-1.55] | 0.90 [0.60-1.05] | 0.70 [0.67-0.95] | 0.80 [0.66-1.12] |
| **RCT End-points** | | | | | | |
| Norepinephrine duration (h) | 60  [46-108] | 117  [83-132] | 96  [60-126] | 42  [32-129] | 132  [108-174] | 108  [53-195] |
| Norepinephrine quantity (µg/kg) | 1222  [1159-2091] | 2082  [1582-3643] | 1788  [1187-3117] | 1000  [683-6617] | 1755  [1560-3849] | 1560  [1091-4896] |
| Septic Shock (%) | 1 (11%) | 4 (33%) | 5 (24%) | - | - | - |
| Infections per patient | 2 [1-3] | 3 [2-3] | 2 [1-3] | 2 [2-3] | 2 [2-3] | 2 [2-3] |
| Skin grafting per patient | 6 [4-7] | 9 [5-11] | 6 [4-10] | 7 [5-7] | 3 [3-4] | 4 [3-7] |
| Stay in ICU (days) | 63 [34-78] | 74 [61-96] | 68 [49-96] | 51 [36-65] | 38 [30-40] | 38 [22-42] |
| Stay in hospital (days) | 75 [65-92] | 93 [66-114] | 85 [65-110] | 70 [46-75] | 38 [30-42] | 42 [26-64] |
| Death before D28 | 1 (11%) | 0 (0%) | 1 (5%) | 1 (34%) | 1 (34%) | 2 (34%) |

**Figure S1 Norepinephrine duration for non-responders patients treated with low-dose hydrocortisone and placebo.**

21 patients were nonresponders to the corticotropin test. Nine nonresponder patients were treated with low-dose hydrocortisone (Corticosteroids, grey boxes) and twelve nonresponder patients were treated with placebo (Placebo, open boxes). Boxplots and individual values of norepinephrine duration (in hours) in low-dose hydrocortisone and placebo-treated groups are presented. Differences between groups were evaluated by using Mann Whitney test.


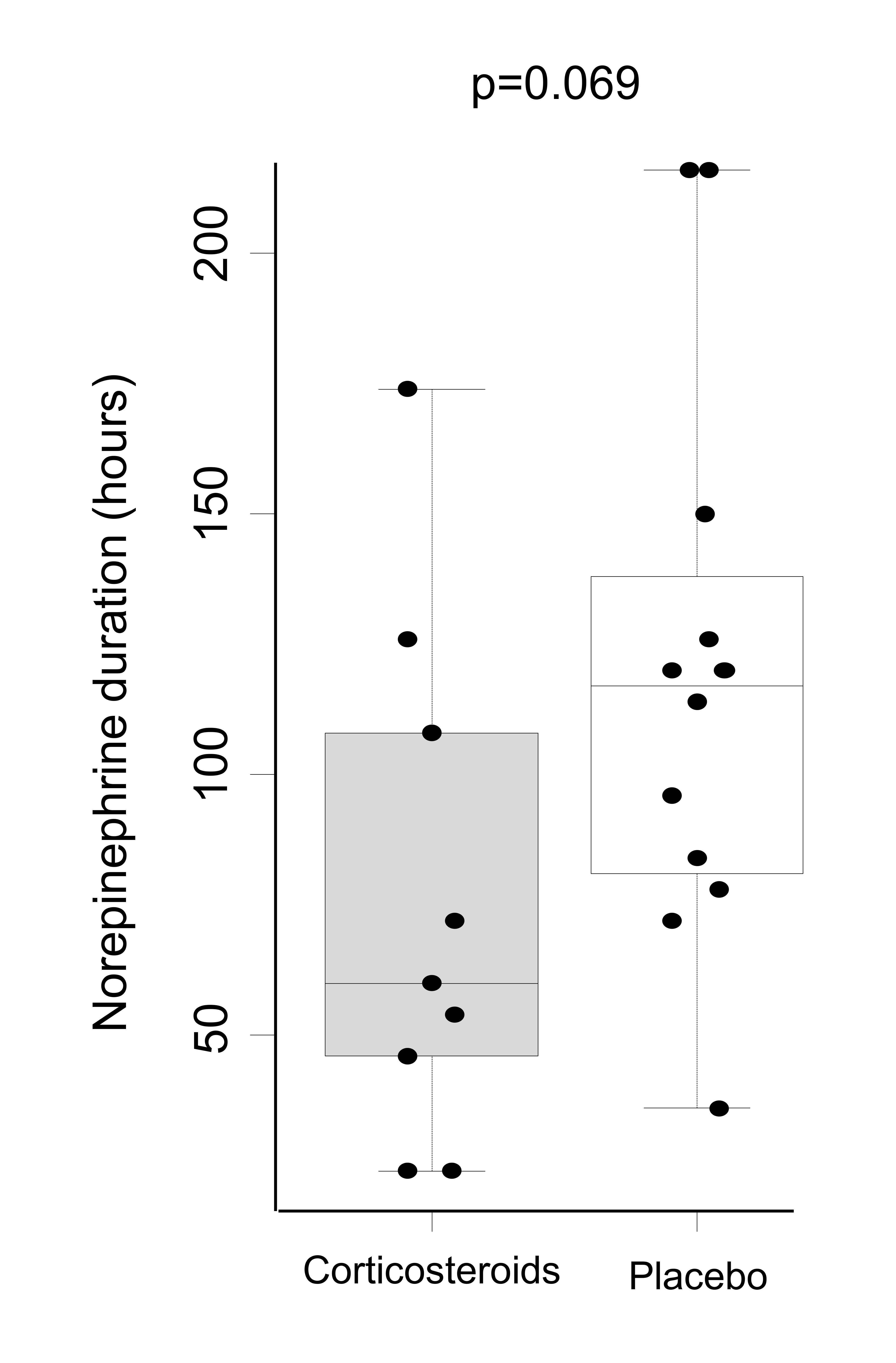

Supplement: Additional file 1: Table S1. — Individual demographic and clinical characteristics of the 32 burn patients. Table S2. Demographic and clinical characteristics of the burn patients with or without RAI. Figure S1. Norepinephrine duration for nonresponder patients treated with low-dose hydrocortisone and placebo. [file 13054_2015_740_MOESM1_ESM.doc]
